# Validation of the pediatric refractory septic shock definition: post hoc analysis of a controlled trial

**DOI:** 10.1186/s13613-021-00822-8

**Published:** 2021-02-10

**Authors:** Luc Morin, Karthik Narayanan Ramaswamy, Muralidharan Jayashree, Arun Bansal, Karthi Nallasamy, Pierre Tissieres, Sunit Singhi

**Affiliations:** 1 Pediatric Intensive Care Unit, Bicêtre Hospital, AP-HP Paris-Saclay University, Le Kremlin-Bicêtre, France; 2 Division of Pediatric Intensive and Emergency Care, Department of Pediatrics, Advanced Pediatrics Centre, Post Graduate Institute of Medical Education and Research, Chandigarh, India; 3 Institute of Integrative Biology of the Cell, CNRS, CEA, Paris Saclay University, Gif-sur-Yvette, France

**Keywords:** Sepsis, Refractory shock, Cardiac dysfunction, Score

## Abstract

**Background:**

The European Society of Pediatric and Neonatal Intensive Care (ESPNIC) developed and validated a definition of pediatric refractory septic shock (RSS), based on two septic shock scores (SSS). Both bedside SSS (bSSS) and computed SSS (cSSS) were found to be strongly associated with mortality. We aimed at assessing the accuracy of the RSS definition on a prospective cohort from India.

**Methods:**

Post hoc analysis of a cohort issued from a double-blind randomized trial that compared first-line vasoactive drugs in children with septic shock. Sequential bSSS and cSSS from 60 children (single-center study, 53% mortality) were analyzed. The prognostic value of the ESPNIC RSS definition was tested for 28-day all-cause mortality.

**Results:**

In this septic shock cohort, RSS was diagnosed in 35 patients (58.3%) during the first 24 h. Death occurred in 30 RSS patients (85.7% mortality) and in 2 non-RSS patients (8% mortality), OR = 60.9 [95% CI: 10.5–676.2], *p* < *0.001* with a median delay from sepsis onset of 3 days [1.0–6.7]. Among patients diagnosed with RSS, the mortality was not significantly different according to vasopressors randomization. Diagnosis of RSS with bSSS and cSSS had a high discrimination for death with an area under the receiver operating curve of 0.916 [95% CI: 0.843–0.990] and 0.925 [95% CI: 0.845–1.000], respectively. High prognostic accuracy of the bSSS was found in the first hours following intensive care admission. The best interval of prognostication occurs after the 12th hour following treatment initiation (AUC 0.973 [95% CI: 0.925–1.000]).

**Conclusions:**

The ESPNIC refractory septic shock definition accurately identifies, within the first 6 h of septic shock management, children with lethal outcome.

## Background

Septic shock, defined as fluid-refractory sepsis with organ dysfunction encompasses various clinical and pathophysiological entities. The Surviving Sepsis Campaign [1] and the American College of Critical Care Medicine guidelines for Hemodynamic Support in Neonates and Children [2] issued guidelines for patients with sepsis including the subset of patients who are unresponsive to vasopressors and labeled as having refractory septic shock (RSS). RSS is described as a circulatory failure due to septic cardiomyopathy [3, 4] with or without vasoplegia [5]. Survival observed following short-term circulatory support with newer vasoactive agents or extra-corporeal life support (ECLS) is an indicator that RSS is potentially reversible [6]. To maximize the impact of these rescue therapies, a robust tool for early detection of RSS was required. The European Society of Pediatric and Neonatal Intensive Care (ESPNIC) defined RSS as the association of high blood lactate with high vasopressor doses and/or myocardial dysfunction [7]. This definition based on two septic shock scores (SSS) showed excellent discriminative power and suggested that both bedside (bSSS) and computed (cSSS) septic shock scores are powerful and potentially useful tools to categorize severity and compare patients in future interventional randomized multicenter studies on septic shock in children. Nevertheless, this RSS definition was established on retrospective data from European and Australian centers and a prospective validation is mandatory as part of the validation process [8]. Furthermore, it may not have been representative of the regional diversity of critically ill children case mix. There is substantial data outlining the differences in sepsis incidence and etiology between regions, with increased incidence of immunodepressed patients (e.g., oncology, solid organ transplantation) in high-income countries in contrast to low- and middle-income countries [9, 10]. In addition, variability in sepsis management may occur and potentially impact sepsis outcome [10]. In order to overcome these limitations, we evaluated the validity of the ESPNIC RSS definition and SSS scores in a post hoc analysis of a double-blind randomized trial aimed at comparing first-line vasoactive drugs in children with septic shock in India [8].

## Material and methods

### Patients

All patients (*n* = 60) included in the double-blind randomized trial at the Advanced Paediatrics Center, Post Graduate Institute of Medical Education and Research, Chandigarh, India, were included in the post hoc analysis. The study protocol has been previously described [8]. In summary, this was a monocentric randomized controlled trial on septic shock children comparing dopamine to epinephrine as first-line vasoactive agents. Patients aged between 3 months and 12 years were enrolled if they had fluid-refractory septic shock and admitted in pediatric intensive care unit between July 2013 and December 2014. The study was registered (CTRI/2014/02/004393) and a local institutional review board approved the use of the database for this study and waived the need for informed consent.

### Data

Patients baseline characteristics including gender, age, Pediatric Risk of Mortality (PRISM) III and Pediatric Logistic Organ Dysfunction (PELOD) score, blood lactate, central venous oxygen saturation (ScvO_2_), vasoactive-inotropic score (VIS) as well as the occurrence of severe acute respiratory distress syndrome (ARDS) defined according to Berlin definition [11] and use of renal replacement therapy were analyzed. There was no missing data, as analyzed items were part of the main outcome measurements from the original study [8]. Both bSSS and cSSS were calculated every 6 h for the first 24 h. The bSSS is a 0 to 5 points score with 1 point for a lactate value above 8 mmol/L or increased > 1 mmol/L after 6 h of management, 1 point for a VIS > 200 and 3 points for the presence of a severe cardiomyopathy defined by a cardiac index < 2.2L/min.m^2^ or a left ventricle ejection fraction < 25%. The cSSS is calculated with: cSSS = 1.1^blood lactate value^ + 1.001^VIS^ + 18 (in the presence of a severe cardiomyopathy). The impact of the interventional group (dopamine or epinephrine) was considered in the analysis. Primary outcome was 28 days all-cause mortality.

### Statistical analysis

Continuous variables were tested for normality with Kolmogorov–Smirnov test and compared with Student’s t test or Mann–Whitney test, as appropriate. Non-continuous variables were tested with Chi-squared test or Fisher’s exact test, as appropriate. Data were described as frequencies and percentages, means and standard deviations or median and interquartile range (IQR), as appropriate. Multivariable Cox’s regression with backward-stepwise method was performed having as outcome 28 days all-cause mortality. Covariates inserted in the models were PRISM III and computed SSS. We measured the discrimination of each score using the area under the receiver operating characteristic curve (AUROC) and expressed values with its 95% confidence interval (CI). The best thresholds for these scores were obtained with the calculation of sensitivity, specificity, positive and negative predictive values and the Youden’s index (Y = sensitivity + specificity − 1). The DeLong test was used to compare the AUROC of both scores. Survival of RSS patients according to both scores have been evaluated by Kaplan–Meier curves and these latter have been tested using log-rank test. A *p* value of less than 0.05 was considered statistically significant. Statistical analyses were performed with SPSS 19.0.0 (SPSS, Chicago, IL) software.

## Results

**Fig. 1:**
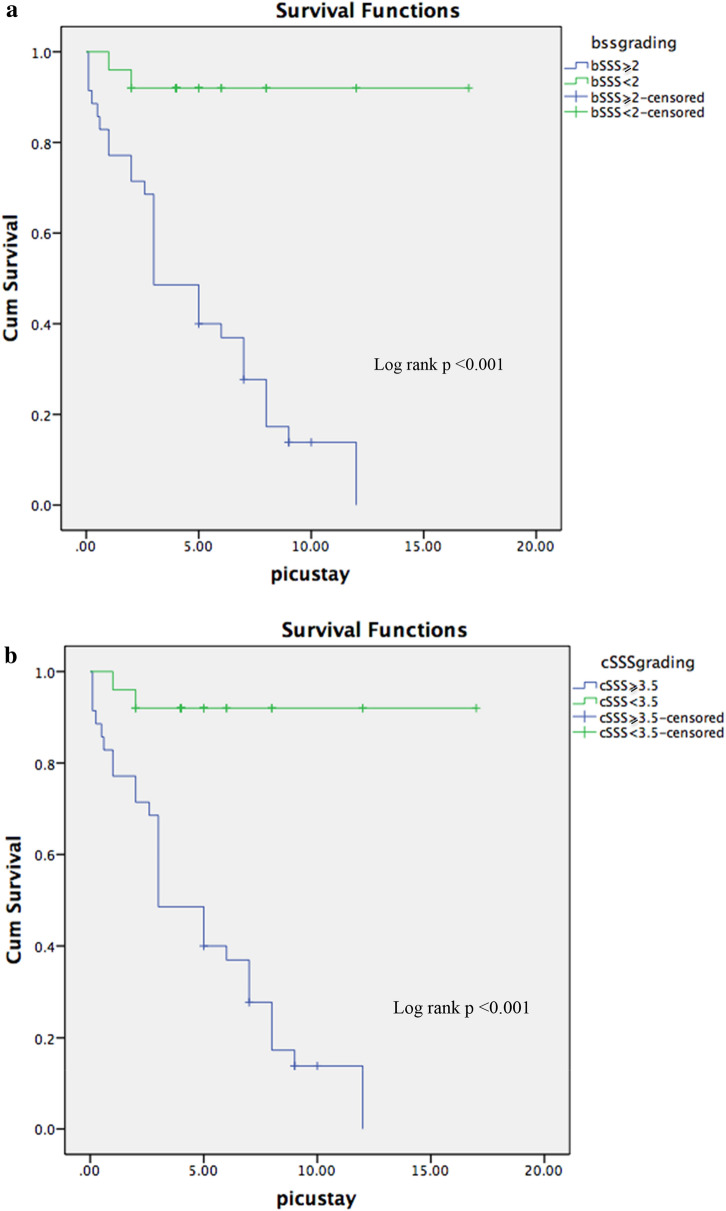
**a** Survival curve of refractory septic shock patients diagnosed with a bedside septic shock score ≥ 2. **b** Survival curve of refractory septic shock patients diagnosed with a computed septic shock score ≥ 3.5

**Fig. 2:**
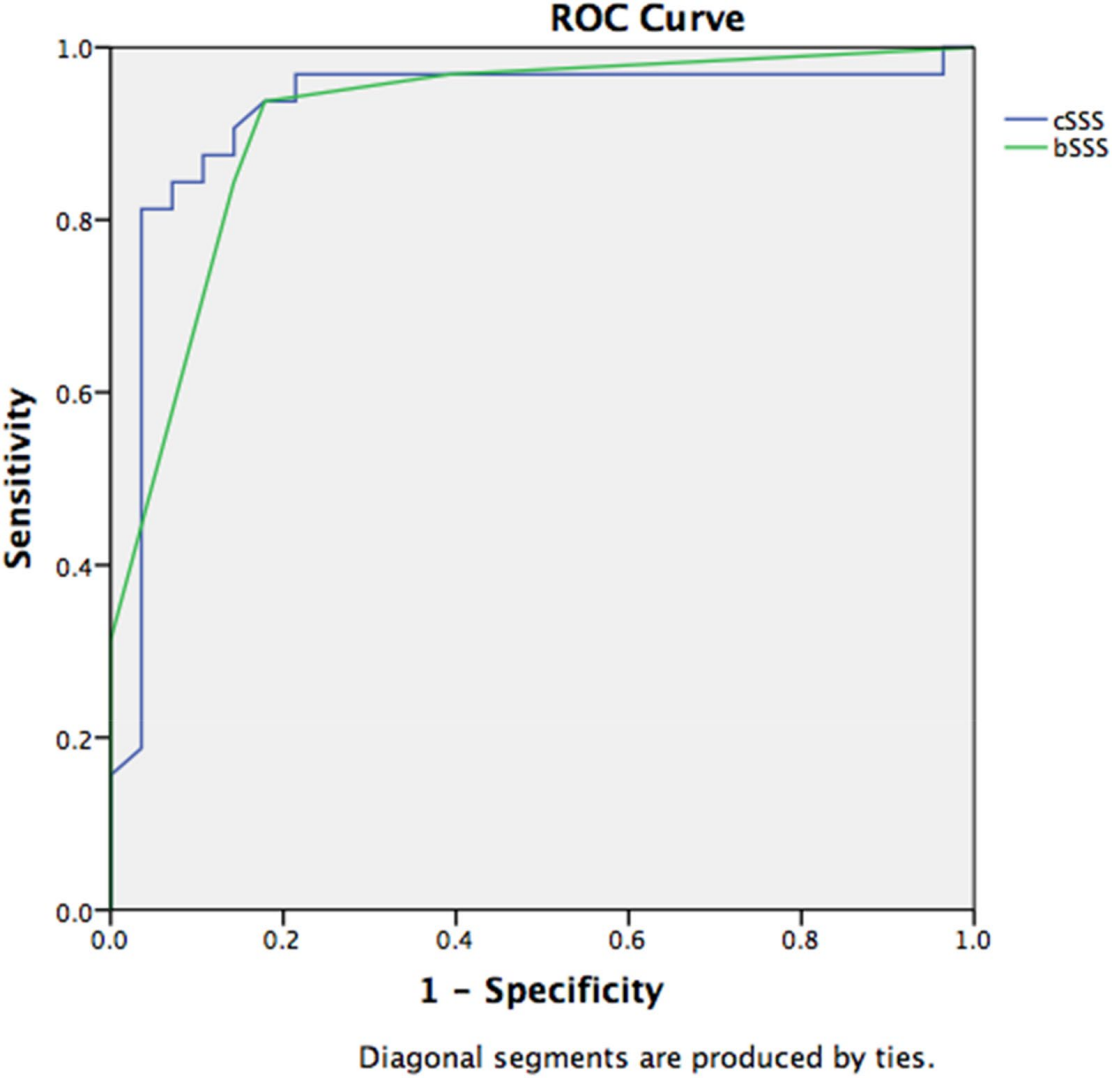
Receiver operating curve (ROC) and area under the ROC for mortality of bedside Septic Shock Score (bSSS) calculated in the first 24 h of admission for septic shock

**Fig. 3:**
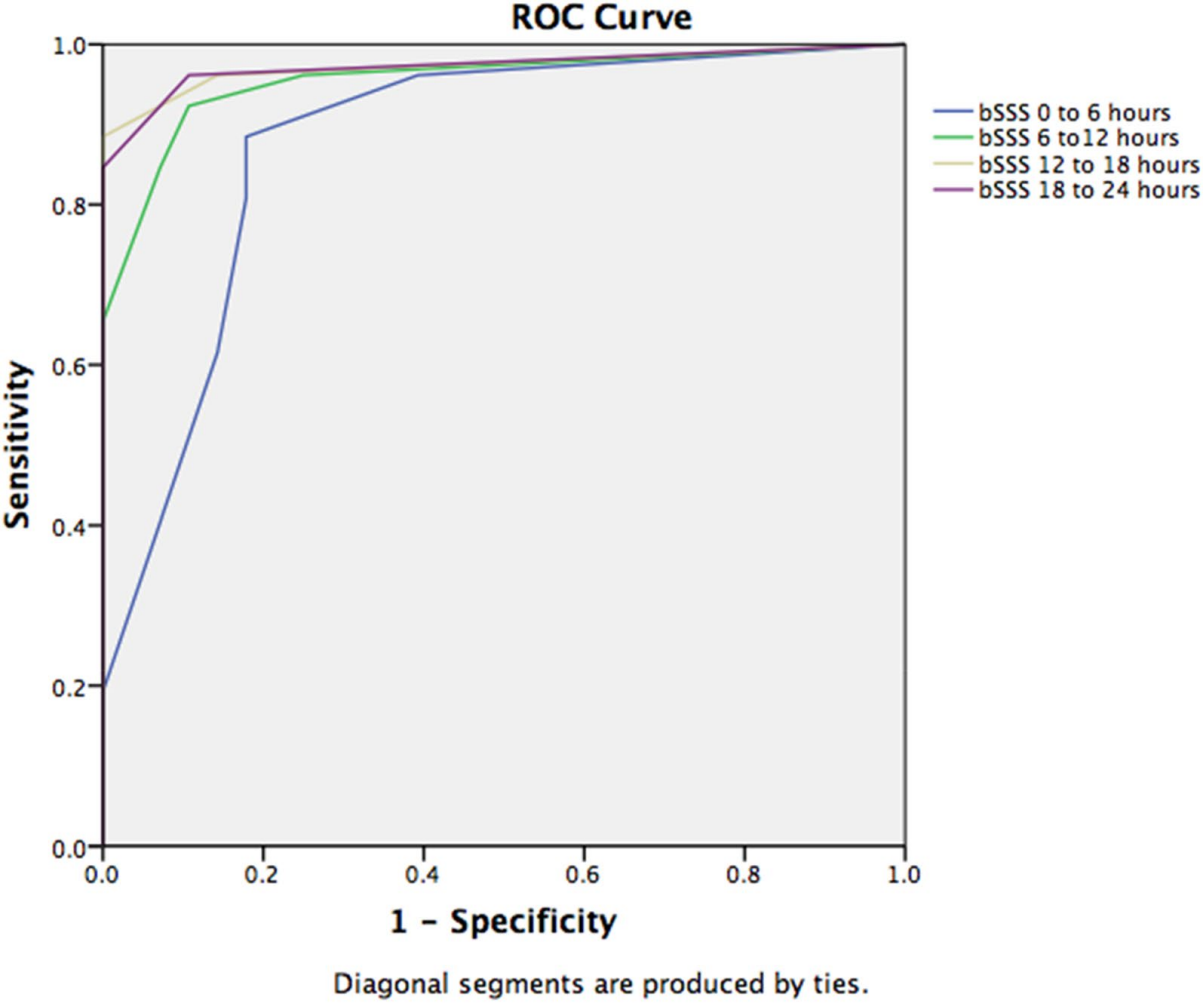
Receiver operating curve (ROC) and area under the ROC for mortality of bedside Septic Shock Score (bSSS) calculated in the first 24 h following admission for septic shock

Of the 210 patients diagnosed with sepsis and screened, 61 children were eligible for enrollment. One parent refused consent, 29 children were randomized to epinephrine group and 31 to dopamine group. All the enrolled subjects completed the study, and were followed up to 28 days. Patients in the epinephrine group achieved earlier resolution of fluid-refractory hypotensive shock in children as compared to dopamine. The baseline characteristics are presented in Table 1. A RSS was diagnosed in 35 patients (58.3%) with a bSSS ≥ 2 or a cSSS ≥ 3.5. Mortality in the RSS patients (*n* = 30/35, 85.7% mortality) was higher than in non-RSS patients (*n* = 2/25, 8% mortality), OR = 60.9 [95% CI: 10.5–676.2], *p* < *0.001*. The survival curves of the patients diagnosed with RSS are presented in Fig. 1a and b. There was no significant difference in discrimination of mortality between bSSS and cSSS with, respectively, an AUROC of 0.916 [95% CI: 0.843–0.990] and 0.925 [95% CI: 0.845–1.000] (Fig. 2). The best binary performance for bSSS was ≥ 2 for bSSS (Youden’s index = 0.76) and > 20,5 for cSSS (Youden’s index = 0.78). A bSSS ≥ 2 was associated with an in-hospital mortality of 50% [positive LR: 5.25, 95% CI = 2.4–11.7], a sensitivity of 93.7% [95% CI: 79.2–99.2] and a specificity of 82.1% [95% CI: 63.1–93.9]. A cSSS > 20.5 was associated with an in-hospital mortality of 93.1% [positive LR: 22.7, 95% CI: 3.3–157], a sensitivity of 81.2% [95% CI: 63.6–92.8] and a specificity of 96.4% [95% CI: 81.7–99.9]. The measure of bSSS between the 12th and 24th hour following septic shock diagnosis was the most discriminative for in-hospital mortality with an AUROC of 0.973 [95% CI: 0.924–1.000], compared to 0 to 6th hours 0.876 [95% CI: 0.780–0.972], *p* = *0.011* (Fig. 3).

**Table 1:** Baseline characteristics

	All patients (*n* = 60)	No RSS (*n* = 25)	RSS (*n* = 35)	*P* value
Age (years)	5.5 (1.0–8.3)	7.0 (4.0–10.2)	1.75 (0.78–1.2)	0.015
Sex female (%)	30 (50%)	11 (44%)	19 (54.3)	0.601
PRISM III at admission	20 (13.5–28.7)	15 (10–18.5)	23 (20–30.5)	< 0.0001
PELOD at admission	32 (12–42)	12 (10.7–31.2)	33 (31–42)	< 0.0001
Community acquired infection	56 (93.3%)	25 (100%)	31 (88.6%)	0.133
Healthcare-associated infection	4 (6.7%)	0	4 (11.4%)	
Systemic infection	26 (43.3%)	16 (64%)	10 (28.5%)	0.008
Pneumonia	14 (23.3%)	5 (20%)	9 (25.7%)	0.761
Use of mechanical ventilation	47 (78.3%)	15 (60%)	32 (91.4%)	0.005
Use of epinephrine as first line	29 (48.3%)	15 (60%)	14 (40%)	0.190
Use of dopamine as first line	31 (51.7%)	10 (40%)	21 (60%)	
Use of dobutamine or milrinone	39 (65%)	11 (44%)	27 (77.1%)	0.014
Use of hydrocortisone	44 (73.3)	14 (56%)	30 (85.7%)	0.017
Use of RBCs transfusion	30 (50%)	18 (72%)	14 (40%)	0.019
Use of RRT	24 (40%)	2 (8%)	22 (62.9%)	< 0.0001
Severe ARDS	17 (28.3%)	3 (12%)	14 (40%)	0.022
Cardiac arrest	4 (6.7%)	0	4 (11.4%)	0.133
Maximal blood lactate (mmol/L)	4.3 (2.6, 6.8)	2.8 (2.4–3.8)	5.5 (3.8–8.9)	< 0.0001
Lactate increase	34 (56.7%)	7 (28%)	27 (79.4%)	< 0.0001
ScvO2 < 70%	52 (86.7%)	20 (80%)	32 (91.4%)	0.259
Myocardial dysfunction on cardiac ultrasound	35 (58.3%)	0	35 (100%)	< 0.0001
Delay from septic shock onset to death	3 (1.0–6.7)	1.5 (1.0–2.0)	3.0 (1.0–7.0)	0.327
Bedside septic shock score (bSSS)	4.0 (0–4.0)	0 (0–1)	4 (4–5)	< 0.0001
Computed septic shock score (cSSS)	20.4 (2.3–21.5)	2.4 (2.3–2.4)	21.4 (20.7–22.3)	< 0.0001
Number of days in PICU	5 (2.7–7.0)	5 (4–7)	3 (2–7)	0.246
PICU mortality	32 (53.3%)	2 (8%)	30 (85.7%)	< 0.0001

Values are expressed as means (standard deviations) or median (interquartile range), as appropriate or number (percent)

*PRISM* Pediatric Risk of Mortality, *PELOD* Pediatric Logistic Organ Dysfunction, *RBC* red blood cell, *ARDS* acute respiratory distress syndrome, *RRT* renal replacement therapy, *ScvO2* central venous oxygen saturation, *PICU* pediatric intensive care unit, *b-/c-SSS* bedside-/computed-Septic Shock Score

We did not find any effect of the group of interventions on the computed score (epinephrine group cSSS = 3.22 [95% IC: 2.32–21.53] and dopamine group cSSS = 20.68 [95% CI: 2.41–21.47], *p* = *0.242*) or the bedside score (epinephrine group bSSS = 1 [95% CI: 0–4] and dopamine group bSSS = 4 [95% CI: 0–4], *p* = *0.281*). Among patients diagnosed with RSS (bSSS > 2 or cSSS > 3.5), the mortality was not significantly different according to randomization to epinephrine (*N* = 13/14 death, 92.9% mortality) or dopamine (*n* = 17/21, 81% mortality), RR = 1.15 [95% CI: 0.89–1.48], *p* = *0.627*. We compared the association of the SSS and PRISM III to mortality using a backward logistic regression and found an independent association with PRISM III (exp(B) = 1.77, 95% CI: [1.11–2.81], *p* = 0.015) and cSSS (exp(B) = 1.495, 95% CI: [1.07–2.08], *p* = *0.018*). The bSSS was not tested as it was not developed as a linear score, but a quick tool to assess patients at the bedside.

## Discussion

This study showed that the bedside and computed septic shock scores discriminated the most severe cases in a prospective cohort from India and confirmed the accuracy of these scores. Patients diagnosed in RSS had a very high mortality. While the presence of a severe myocardial dysfunction is not mandatory for the diagnosis of RSS, it was present in all patients in this study. This point reinforces the importance of septic cardiomyopathy in the pathophysiology of RSS. El Nawawy et al*.* demonstrated in a small monocentric cohort the added value of myocardial and hemodynamic evaluation with echocardiography and confirmed the high incidence of myocardial dysfunction in pediatric septic shock [12, 13]. The vasoactive-inotropic score used in the SSS is validated in pediatric sepsis as a marker of severity. Interestingly, MacIntosh et al*.* found a similar pattern of evolution of the VIS with a best correlation with morbidity after the 12th hour of care [14]. Meanwhile, the addition of blood lactate and myocardial dysfunction in the SSS may explain the excellent discrimination power as early as within the first 6 h of management. Blood lactate with a cut-off value of 4 mmol/L was associated with mortality in a large cohort of pediatric sepsis patients [15]. Despite having a higher lactate cut-off value of 8 mmol/L, the bSSS does not seem to have lower sensitivity than the cSSS. In this study, both bSSS and cSSS identified the same number of RSS patients with similar outcome*.* The high lactate cut-off value in the bSSS effectively selects patients at the highest risk of mortality and with catecholamine-refractory shock. Interestingly, bSSS ≥ 2 cut-off confirmed the one set in the original study [7]. However, cSSS best cut-off is higher in this cohort than in the original. This is due to a strong impact of myocardial dysfunction (+ 18 points) and its elevated incidence in the present cohort where all patients in RSS had myocardial failure. The stronger association of myocardial dysfunction and mortality seen in this study emphasize the heterogeneity in the case mix and management, and further support the use of bSSS that shows to encompass accurately the whole spectrum of RSS.

Our previous study’s main limitation was the uncertainty about the simultaneity of the measure of lactate, VIS and/or the presence of cardiomyopathy. The present study focused on the first 24 h of management, all the measures were associated with discrimination for mortality. Nevertheless, the scores were best predictive of mortality after the first 12th hour of management. This may be explained by the need for the vasopressors to be progressively increased until the VIS cut-off is reached, or the patient deteriorates with the occurrence of septic cardiomyopathy. Timing is essential in RSS diagnosis. An early diagnosis of RSS in the first 24 h associated with a 60% [7] to 85.7% (present study) estimated mortality should be a strong indicator of need for adjunct therapies.

A recent study found that refractory shock is the first cause of mortality in pediatric septic shock patients [16]. This fact emphasizes the need for a definition of RSS, thus, its validation in this study. Despite being a less frequent cause of death, multiple-organ dysfunction syndrome (MODS) can be diagnosed or assessed with several scores such as PELOD 2 and PIM 2. While PIM 2 may only be measured at admission, PELOD 2 can be measured sequentially [17]. However, in our study PELOD was not calculated sequentially. The PRSIM III score, designed to estimate mortality, was found to be independently associated with mortality. Although formerly not evaluating organ failure, PRSIM III score is calculated using many clinical and biochemical criterion surrogated of organ failures. In the RSS definition process, individual organ failures were not retained by pediatric critical care experts preferring quantifiable cardio-circulatory criterion [7]. Our study confirms the importance of cardio-circulatory failure in sepsis severity and prognostication and although not being specifically investigated, a potential hierarchical importance over individual organ failure may be suggested.

The first limitation of this study is that it was a post hoc analysis of a single-center randomized trial. The use of inotrope or vasopressor in the original study was protocolized, which may have affected the increase in the inotropes or vasopressors, hence the VIS and the predictive performances of the SSS in the first hour of management. Nevertheless, the randomization drug was not associated with increase mortality in the multivariable analysis. Secondly, all patients diagnosed with RSS had a severe cardiomyopathy diagnosed with cardiac ultrasound, which is a sufficient criterion for the diagnosis of RSS. Indeed, cardiac ultrasound is questionable due to the subjectivity of the exam and the risk of error for the diagnosis of cardiac failure due to the presence of hypovolemia. Yet, this risk for over-diagnosis of RSS appears unlikely when considering the good performance of the scores and the high mortality in the RSS group. Thirdly, most scores used or currently discussed in the question of sepsis and septic shock diagnosis were unavailable in our cohort, hence we could not compare their performance with the SSS.

This study’s strengths are the homogeneous and well-characterized cohort of patients with septic shock with timely measured SSS. It is to our knowledge the first prospective study to evaluate sequentially during the first 24 h the prognosis performances of these scores in pediatric septic shock patients. This study provides clear evidence that RSS is a specific entity responsible for most deaths in pediatric septic shock.

## Conclusion

This post hoc prospective single-center study validates septic shock scores used to diagnose refractory septic shock, showing excellent discriminatory power for mortality in the first hours of management. Future studies should explore the application of this definition in a multicentric prospective cohort of patients and investigate the benefits obtained from adjunct therapies dedicated to the patients with the highest risk of death.

## Data Availability

All materials are available upon request to Pr. Sunit Singhi at sunitcsinghi@gmail.com.

## Abbreviations

ESPNIC: European Society of Pediatric and Neonatal Intensive Care
RSS: Refractory septic shock
b-/c-SSS: Bedside/computed Septic Shock Score
ECLS: Extra-corporeal life support
PRISM: Pediatric Risk of Mortality
MODS: Multiple-organ dysfunction syndrome
PELOD: Pediatric Logistic Organ Dysfunction
PIM: Pediatric Index of Mortality
VIS: Vaso-Inotropic Score
LR: Likelihood ratio
CI: Confidence interval
